# South Texas Medical Association

**Published:** 1897-06

**Authors:** E. S. Ferguson

**Affiliations:** Secretary


### South Texas Medical Association—Second Annual Meeting—Galveston.

The second semi-annual meeting of the South Texas Medical Association, which met in Galveston on Friday, May 14th, was a decided success—about fifty physicians being in attendance.

All the papers read were of a superior order, and elicited enthusiastic discussion.

The morning session opened at 10 o'clock in the Chamber of Commerce rooms, in the Tremont Hotel, Dr. Bat Smith, of Wharton, presiding.

Dr. W. F. Starley, Jr., of Galveston, delivered an eloquent address of welcome, which, in the absence of Dr. Cunningham, appointed to reply, was responded to in a happy manner by Dr. B. F. Calhoun, of Beaumont.

The first paper, "Ligation of Common Carotid Artery," by Dr. R. T. Morris, of Houston, gave a report of several very interesting cases, and recommended the use of animal ligatures, preferrably kangaroo tendon. The paper was discussed by Drs. H. A. West, O. L. Norsworthy, Cannon and Thompson.

"Wound-Grafting with Tissues from the Lower Animals," by Dr. Trueheart, of Galveston. The author presented a case, showing an excellent result of grafting on the hand by tissues from along the mammary glands of young dogs, antiseptic precautions being strictly observed. Discussed by Drs. Morris, Keiler, Thompson and Norsworthy.

"Limpomata," by Dr. Sofie Herzog, of Brazoria, the only lady in attendance. The doctor's paper was well received, and showed her to be perfect master of her subject. She reported several cases where deep injections of iodine had accomplished the happiest results, and recommended the treatment to the profession. The paper was highly complimented, and discussed by Drs. Smith, Keiler and Thompson.

"The Treatment of Inevitable Abortion," by Dr. R. W. Knox, of Houston, closed the morning session, and was an interesting paper. The doctor recommended very highly the use of the tampon, and called special attention to the many diseased conditions caused by abortions and miscarriages. He urged the profession to study the subject carefully. Discussed by Drs. Paine and West.

The afternoon session met at 2:30 in the college building.

"Operative Interference in Tumors of the Upper Jaw, Nose and Pharynx," by Dr. J. E. Thompson, of Galveston. This was an excellent paper, the doctor reporting some very interesting cases, and describing some unique operations. Discussed by Drs. Keiler and Knox.

"Anatomy of the Kidney," by Dr. Bat Smith, of Wharton, was next read, showing close study into this intricate organ. Discussed by Dr. Keiler.

"Consideration of Catarrhal Deafness in the Young," by Dr. J. A. Mullen, of Houston. The doctor pointed out the necessity of the care of the nose and throat in early infancy in order to prevent this much dreaded trouble. Discussed by Drs. Schenk, Knox, Reuss.

"Etiology and Treatment of Dysentery," by Dr. West of Galveston. The paper brought forth a warm discussion by Drs. Price, Cook, Reuss and Smith.

The Association then went into business session. Beumont was selected as the next place of meeting; time, December 28th. Dr. J. S. Price, chairman of committee of arrangements.

On assembling at 7:30 p. m. Dr. Seth Morris, of Galveston, exhibited an X-Ray machine, which was much enjoyed.

Dr. B. F. Calhoun, of Beaumont, read a paper on "Contraindications to the Use of the Coal Tar Derivatives," and condemned the indiscriminate use of these drugs as at present prescribed as both harmful and dangerous. Discussed by Drs. Cerna, West, Morris, Price, Reuss, Sampson and Shearer, closed by Dr. Calhoun.

Dr. J. H. Sampson presented a paper on "Some Practical Observations in Pelvic Operation." A number of interesting operations were outlined, the author recommending the vaginal route as the most scientific. Discussed by Drs. Kennedy, A. F. Sampson, Moore. Closed by Dr. Sampson.

The essay on the "Hypordermic Use of Iron," by Dr. David Cerna, of Galveston, was particularly interesting, and brought forth many happy comments. In pernicious anemia and the anemia following the wasting diseases, the author stated could be best combatted by the subcutaneous method of administering irons. Citrate of iron and ammonia and citrate of iron and manganese were the preparations recommended. Discussed by Drs. Calhoun, Norsworthy, J. H. Sampson, Morris, Reuss, Moore. Closed by Dr. Cerna.

Dr. O. L. Norsworthy closed the session with a paper on "The Phonendoscope and Its Use," giving a history of the instrument, and pointing out its use in making accurate diagnoses, recommending it particularly in obstetrical work. Discussed by Drs. Price, Smith and Cerna. Closed by Dr. Sampson.

The Association adjourned to meet on December 28th in Beaumont.

E. S. Ferguson, M. D., Secretary.

